# INTRAINDIVIDUAL VARIABILITY MODIFIES COGNITIVE TRAINING IN COGNITIVELY VULNERABLE OLDER ADULTS WITH HIV

**DOI:** 10.1093/geroni/igae098.2783

**Published:** 2024-12-31

**Authors:** David Vance, Andres Azuero, Julie Schexnayder, Frank Puga, Jun Byun, Chunhong Xiao, Dara James, Pariya Wheeler

**Affiliations:** University of Alabama at Birmingham, Birmingham, Alabama, United States; University of Alabama at Birmingham, Birmingham, Alabama, United States; University of Alabama at Birmingham, Birmingham, Alabama, United States; University of Alabama at Birmingham, Birmingham, Alabama, United States; University of Alabama at Birmingham, Birmingham, Alabama, United States; University of Alabama at Birmingham/School of Nursing, Birmingham, Alabama, United States; Arizona State University, Tempe, Arizona, United States; University of Alabama at Birmingham, Birmingham, Alabama, United States

## Abstract

Cognitive Intra-Individual Variability (IIV), the fluctuation in cognitive performance within an individual, often stems from compromised cognitive control due to brain pathology. In cognitive aging studies, cognitive IIV has been shown to predict cognitive decline 3 years later, dementia, and mortality. Those with HIV-Associated Neurocognitive Disorder (HAND) frequently exhibit heightened cognitive IIV; this is concerning as the HIV population has become an aging epidemic (i.e., by 2030 70% of people with HIV will be 50 and over). This study investigated whether computerized Speed of Processing (SOP) training could mitigate cognitive IIV and whether baseline cognitive IIV influenced the impact of SOP training on individuals with HAND or borderline HAND. In a 3-group experimental design, 216 middle-aged and older individuals living with HIV (PLWH) and HAND were randomly assigned to: 1) 10 hours of SOP training (n=70); 2) 20 hours of SOP training (n=73); or 3) 10 hours of Internet Navigation Control Training (n=73). Participants underwent a seven-domain cognitive battery at baseline, posttest after training, and at 1 and 2-year follow-ups, with the coefficient of variation (CoV) representing cognitive IIV dispersion. Linear mixed-effect models did not reveal an obvious pattern of SOP training effects on cognitive IIV. Albeit, a three-way interaction tests between group, time, and baseline IIV CoV [F(6, 453.96)=1.74, P= 0.11], and group, time, and baseline IIV CoV median split indicator [F(6, 467.84)=2.024, P= 0.061] posited potential moderation effects. While SOP training did not directly enhance cognitive IIV, its effects may be influenced by individual baseline cognitive IIV.

